# Characteristics of the cytoplasmic halo during fertilisation correlate with the live birth rate after fresh cleaved embryo transfer on day 2 in minimal ovarian stimulation cycles: a retrospective observational study

**DOI:** 10.1186/s12958-021-00859-1

**Published:** 2021-11-26

**Authors:** Kenji Ezoe, Tetsuya Miki, Tadashi Okimura, Kazuo Uchiyama, Akiko Yabuuchi, Tamotsu Kobayashi, Keiichi Kato

**Affiliations:** Kato Ladies Clinic, 7-20-3 Nishishinjuku, Shinjuku-ku, Tokyo, 160-0023 Japan

**Keywords:** Cleaved embryo transfer, Cytoplasmic halo, In vitro fertilisation, Intracytoplasmic sperm injection, Live birth, Pronucleus

## Abstract

**Background:**

Information regarding the influence of cytoplasmic events during fertilisation on the clinical outcome remains limited. The cytoplasmic halo is one of these events. A previous study that used time-lapse technology found an association of the presence and morphokinetics of the cytoplasmic halo with cleavage patterns, development to the blastocyst stage, and the ongoing pregnancy rate after blastocyst transfer. Therefore, the cytoplasmic halo may be a useful predictor of the pregnancy outcome after cleaved embryo transfer. This study evaluated the ability of the cytoplasmic halo to predict a live birth after fresh cleaved embryo transfer on day 2, and sought to identify factors potentially influencing the presence and morphokinetics of the halo.

**Methods:**

A total of 902 embryos cultured in the EmbryoScope+® time-lapse system and subjected to single fresh cleaved embryo transfer were retrospectively analysed. The presence and duration of a cytoplasmic halo were annotated. The initial positions of the pronuclei were also observed. The correlation between the cytoplasmic halo and live birth was evaluated and the association of the cytoplasmic halo with patient, cycle, and embryonic characteristics was determined.

**Results:**

Absence of a cytoplasmic halo was associated with a significant decrease in the likelihood of a live birth after fresh cleaved embryo transfer. Prolongation of the halo, especially the duration of central repositioning of cytoplasmic granules, had an adverse impact on the live birth rate. The characteristics of the cytoplasmic halo were not affected by the ovarian stimulation method used, female age, the serum steroid hormone level on the day of trigger, or semen quality. However, the cytoplasmic halo was significantly affected by male age, oocyte diameter, and the initial position of the male pronucleus.

**Conclusions:**

Absence or prolongation of the cytoplasmic halo was negatively correlated with the live birth rate after fresh cleaved embryo transfer. The characteristics of the cytoplasmic halo were strongly associated with oocyte diameter, male age, and the initial position of the male pronucleus. These findings indicate that the characteristics of the cytoplasmic halo can be used to select more competent embryos for transfer at the cleavage stage.

**Supplementary Information:**

The online version contains supplementary material available at 10.1186/s12958-021-00859-1.

## Background

Fertilisation is a critical biological event in sexual reproduction and comprises a series of processes [1–4]. After extrusion of a second polar body, the DNA in sperm decondenses and forms a male pronucleus, and the DNA in the oocyte also decondenses and forms a female pronucleus [1, 2]. The male and female pronuclei migrate towards the centre of the ooplasm, where the nuclear envelopes interdigitate and the two pronuclei become invisible [3, 4]. Using time-lapse technology, these transient events during the fertilisation process can be observed in detail, unlike with conventional static observation using stereomicroscopy or inverted microscopy. The timing of nuclear events during the fertilisation process, including appearance and fading of the pronuclei, has been used in clinical embryology to predict pre-implantation development, implantation, and live birth outcomes [5–9]. However, information on the influence of the cytoplasmic events that occur during the fertilisation process on the clinical outcome is still limited. Condensation of organelles, seen as a cytoplasmic halo, is a cytoplasmic event observed during fertilisation [10, 11]. A previous study that used static observation found that the presence of a cytoplasmic halo had an impact on embryo quality and implantation [12]. Furthermore, another study that used time-lapse technology found a significant correlation between the duration of the cytoplasmic halo during fertilisation and embryo quality on day 3 [13]. Our recent study revealed that the presence and morphokinetics of the halo are associated with the cleavage pattern, development to the blastocyst stage, and the ongoing pregnancy rate after single blastocyst transfer [14]. Therefore, it was hypothesised that the presence of a halo and its morphokinetics would be useful predictors of the pregnancy outcome after cleaved embryo transfer. The aims of this study were to evaluate the ability of the cytoplasmic halo to predict the probability of a live birth after fresh cleaved embryo transfer on day 2, and to identify factors potentially influencing the presence and morphokinetics of the halo by assessing their correlation with patient, cycle, and embryonic characteristics.

## Methods

### Patients

The study included 902 women who underwent oocyte retrieval during a natural cycle, a clomiphene citrate (CC)-based minimal stimulation cycle, or a letrozole cycle at Kato Ladies Clinic between March and July 2018. The clinical records of 902 fresh cleaved embryos on day 2 were retrospectively reviewed. Women with recurrent implantation failure (four or more unsuccessful embryo transfers) [15] and cases where surgical retrieval of sperm was required were excluded.

### In vitro fertilisation during a natural cycle, a CC-based minimal ovarian stimulation cycle, and a letrozole cycle

In the in vitro fertilisation (IVF) protocol for a natural cycle, the only pharmacological intervention used was a gonadotropin-releasing hormone agonist for induction of final oocyte maturation. Monitoring consisted of an ultrasound scan and a hormone profile; this was usually initiated on the morning of day 10 and/or 12 according to the length of the patient’s cycle. When the leading follicle reached 18 mm in diameter and the oestradiol level was > 250 pg/mL, ovulation was triggered by nasal administration of the gonadotropin-releasing hormone agonist buserelin (Suprecur Mochida Pharmaceutical Co., Ltd., Tokyo, Japan; or Buserecur, Fuji Pharma Co., Ltd., Tokyo, Japan).

Details of the protocol for minimal stimulation with CC have been reported previously [16, 17]. Briefly, CC (50–100 mg/day; Fuji Pharma Co., Ltd) was administered with an extended regimen from cycle day 3 until induction of final oocyte maturation. Ultrasonographic and hormonal follicle monitoring was initiated on day 8 and performed daily until ovulation was triggered. When the dominant follicle developed to a size larger than 18 mm, ovulation was triggered by buserelin.

For IVF during a letrozole cycle, letrozole (Novartis, Basel, Switzerland) was administered at a dosage of 5 mg/day on days 3–7, and follicular development was monitored by hormone assay and ultrasonography. When the leading follicle reached 18 mm in diameter, ovulation was triggered by buserelin.

Oocyte retrieval was performed at 34–36 h after triggering using a 21-G needle (Kitazato Corporation, Shizuoka, Japan). Cumulus-oocyte complexes were collected, washed, and transferred to human tubal fluid (HTF) medium (Kitazato Corporation) under paraffin oil at 37 °C (gas phase: 5% O_2_, 5% CO_2_, and 90% N_2_) until either conventional IVF 3 h later or denudation in cases of intracytoplasmic sperm injection (ICSI) 4 h after oocyte retrieval [18]. The oocytes were denuded and cultured in HTF medium prior to ICSI.

Sperm samples were collected by masturbation and washed by centrifugation through 70 and 90% density gradients (Isolate; Irvine Scientific, Santa Ana, CA, USA). The prepared sperm samples were cultured in HTF medium at 37 °C (gas phase: 5% O_2_, 5% CO_2_, and 90% N_2_) until use.

### Conventional insemination, intracytoplasmic sperm injection, and embryo culture

For conventional IVF, HTF medium supplemented with 10% serum substitute (Irvine Scientific) was used as the fertilisation medium [18]. Cumulus-oocyte complexes were cultured with sperm (100,000 sperm/mL) at 5% CO_2_ in air at 37 °C. Extrusion of the second polar body was confirmed 5 h after insemination (day 0) following removal of cumulus cells. The oocytes were individually cultured in an EmbryoSlide (Vitrolife, Inc., Göteborg, Sweden), which is suitable for group culture in 180 μL of medium drop (ONESTEP medium; Nakamedical, Inc., Tokyo, Japan) under paraffin oil. In cases of ICSI, the oocytes were inseminated in HEPES-buffered HTF medium [19, 20]. Injected oocytes were immediately placed onto EmbryoSlides and cultured individually in 180 μL of medium under paraffin oil. Embryos were cultured at 37 °C (gas phase: 5% O_2_, 5% CO_2_, and 90% N_2_) in an Embryoscope+ time-lapse incubator (Vitrolife) for 2 days. Fertilisation was observed using EmbryoViewer software (Vitrolife).

### Annotation and assessment of embryo development

**Fig. 1:**
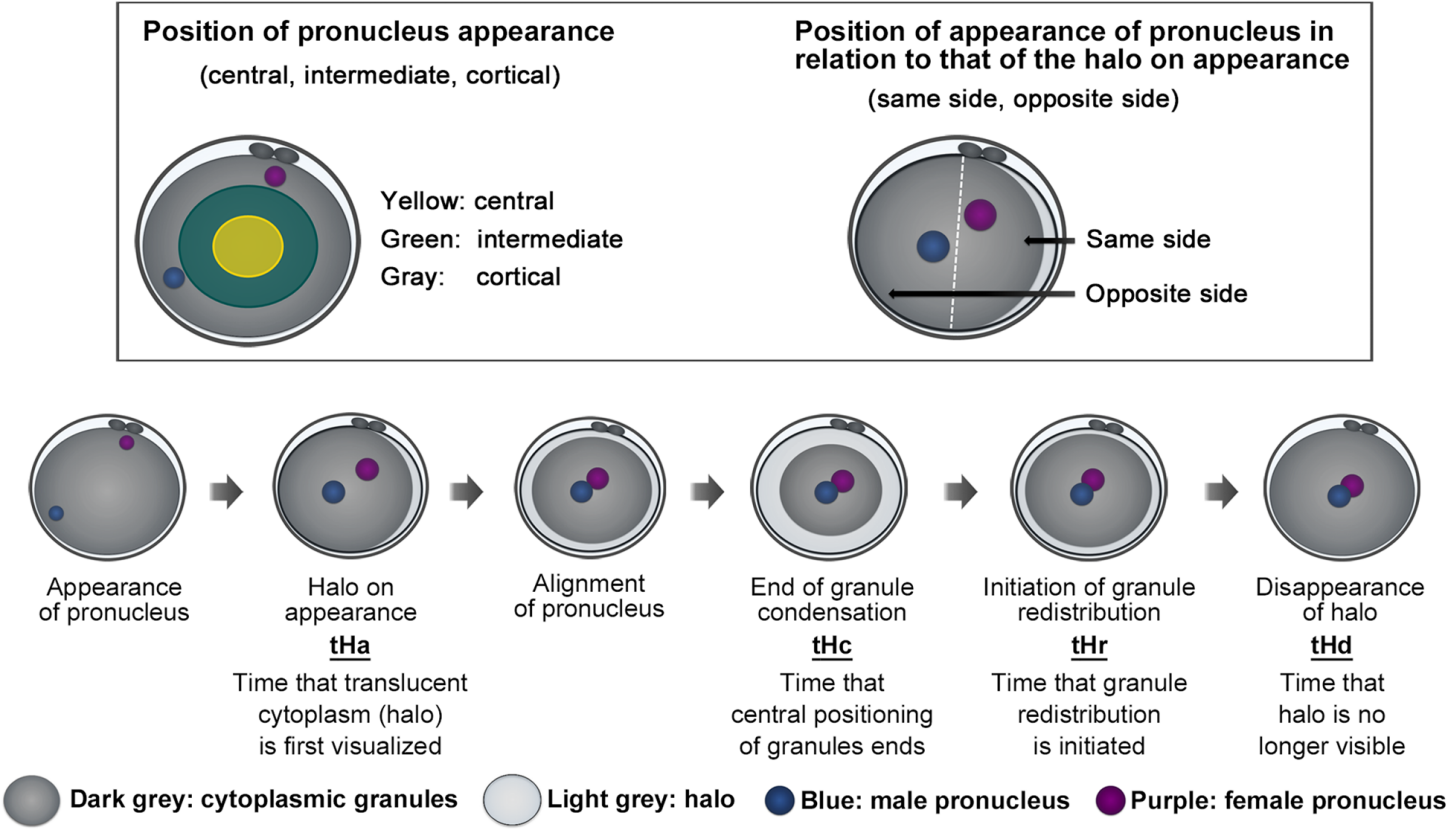
Definition of parameters annotated during fertilisation. The initial positions of the pronuclei were categorised into three groups: central (yellow), intermediate (green), and cortical (grey). The position of the pronucleus in relation to that of the cytoplasmic halo was also annotated (same side vs. opposite side). Dark grey, cytoplasmic granules; light grey, halo; blue, male pronucleus; purple, female pronucleus

Images were taken every 10 min for each embryo in 11 focal planes. Timing of parameters indicating biological events in the embryos were recorded by EmbryoViewer during 2 days of culture, as previously reported [14, 18]. The presence and timing of the intra-cytoplasmic halo phenomenon prior to the first division was also annotated (Fig. 1), as reported previously [14]. Briefly, the time of halo initiation (tHa) was first identified by movement of cytoplasmic granules towards the centre of the oocyte and appearance of peripheral cytoplasmic translucency. After the cytoplasmic granules had moved to the centre and were stationary, the time when centripetal movement ended (tHc) was recorded. After central repositioning, the granules began to move back to the cortex. We recorded tHr as the time when the granules started to redistribute, and tHd as the time when the halo phenomenon disappeared. We also recorded the position of the female/male pronuclei when they appeared in relation to the cytoplasmic halo (i.e., same side vs. opposite side). The pronuclei, which formed cortically and near the site of emission of the second polar body, were annotated using the female pronucleus, as reported earlier [13]. The oocyte diameter was measured thrice at PN break down, and the average of these measurements was used. Cleavage-stage embryos were graded using Veeck’s criteria 42 h after insemination [21]. All annotations in the Embryo Viewer software and conventional morphological grading of embryos were performed by two operators with more than 10 years of experience in embryology, who were blinded to the clinical outcomes.

### Embryo transfer

Single fresh cleaved embryo transfer was performed on day 2 after oocyte retrieval, as previously described [18, 22]. Dydrogesterone (30 mg/day orally) was routinely administered during the early luteal phase after the embryo transfer procedures. If luteal function was inadequate, progesterone was administered intravaginally (Lutinus, Ferring Pharmaceuticals, Saint Prex, Switzerland) until week 9 of pregnancy. Clinical pregnancy was defined by observation of a gestational sac at 5–6 weeks after embryo transfer on an ultrasound scan and ongoing pregnancy by detection of a foetal heartbeat at 7 weeks after embryo transfer. The clinical pregnancy rate, ongoing pregnancy rate, and follow-up data on live births were analysed.

### Statistical analysis

Trends in proportions were analysed using the Cochran–Armitage and Fisher’s exact tests. Continuous variables were compared using the Student’s *t*-test or one-way analysis of variance. The statistical significance of differences was determined by post-hoc analysis using Tukey’s test. The Spearman rank correlation test was used to measure the degree of association between two continuous variables. Logistic regression was used to identify variables that were potentially associated with the pregnancy outcome. Odds ratios and adjusted odds ratios (aORs) are reported with 95% confidence intervals (CIs) for each group. Receiver-operating characteristic (ROC) curve analysis was performed, and the area under the ROC curve was calculated. Multiple linear regression analysis was used to assess the relative importance of the possible predictor variables in the duration of cytoplasmic halo. All statistical analyses were performed using JMP software (SAS Institute Inc., Cary, NC, USA). A *P*-value < 0.05 was considered statistically significant.

## Results

### Cycle and embryonic characteristics

The patient demographics and cycle characteristics are shown in Table 1. During the study period, 902 patients were scheduled for 902 oocyte retrieval/embryo transfers during a natural cycle (13.7%), CC cycle (79.2%), or letrozole cycle (71.4%). Among the 902 embryos transferred, 279 (30.9%) were produced by conventional IVF, and 623 (69.1%) were produced by ICSI (Table S1). The proportion of embryos with a cytoplasmic halo was 96.0% (866/902), and the duration of the cytoplasmic halo (i.e., the time interval [TI] from tHa to tHd) was 14.13 ± 0.09 h (Table S1).

**Table 1:** Association of the cytoplasmic halo with cycle characteristics and clinical outcomes

	Halo-positive	Halo-negative	*P*-value
Patients, n	866	36	
Female age (y)	36.8 ± 0.1	38.2 ± 0.6	0.0517
Male age (y)	39.3 ± 0.2^a^	43.1 ± 1.2^b^	0.0001
Previous embryo transfer cycles, n	0.51 ± 0.01	0.50 ± 0.01	0.8992
Cause of infertility			0.9447
Ovulation, n (%)	30 (3.5)	1 (2.8)	
Tubal factor, n (%)	16 (1.9)	1 (2.8)	
Endometrial factor, n (%)	70 (8.1)	4 (11.1)	
Male factor, n (%)	112 (12.9)	3 (8.3)	
Combined, n (%)	20 (2.3)	1 (2.8)	
Unexplained, n (%)	618 (71.4)	26 (72.2)	
Ovarian stimulation			0.8694
Natural cycle, n (%)	120 (13.9)	4 (11.1)	
Clomiphene citrate cycle, n (%)	685 (79.1)	29 (80.6)	
Letrozole cycle, n (%)	61 (7.0)	3 (8.3)	
Oestradiol level on day of the trigger (pg/mL)	777.6 ± 14.1	717.0 ± 63.4	0.3925
Progesterone level on day of the trigger (ng/mL)	0.4 ± 0.0	0.4 ± 0.0	0.2016
Oocytes retrieved, n	1.9 ± 0.0	1.9 ± 0.1	0.8497
Oocyte diameter	113.5 ± 0.2	113.3 ± 0.6	0.7871
Total sperm number (×10^6^)	173.8 ± 6.1	155.7 ± 21.9	0.5539
Motile sperm number (×10^6^)	93.1 ± 3.9	68.9 ± 10.2	0.2067
Motile sperm rate (%)	50.7 ± 0.6	46.6 ± 3.0	0.1883
Abnormal sperm number (×10^6^)	164.6 ± 5.8	147.4 ± 20.7	0.5477
Abnormal sperm rate (%)	94.8 ± 0.1	94.8 ± 0.3	0.8384
Embryos transferred, n	866	36	
Blastomeres in embryos transferred	5.8 ± 0.1	5.3 ± 0.3	0.0802
Morphological grade based on Veeck’s criteria			
1, n (%)	133 (15.4)	4 (11.1)	0.4866
2, n (%)	250 (28.9)	9 (25.0)	0.6152
3, n (%)	478 (55.2)	21 (58.3)	0.7107
4, n (%)	5 (0.6)^a^	2 (5.6)^b^	0.0009
Clinical pregnancy, n (%)	251 (29.0)	6 (16.7)	0.1087
Ongoing pregnancy, n (%)	215 (24.8)	4 (11.1)	0.0600
Live birth, n (%)	183 (21.1)^a^	2 (5.6)^b^	0.0233
Miscarriage, n (%)	61 (24.3)^a^	4 (66.7)^b^	0.0183

The data are shown as the mean and standard error of mean unless otherwise indicated

*tHa* Time of halo initiation, *tHc* Time when centripetal movement ended, *tHd* Time when the halo phenomenon disappeared, *tHr* Time when the granules started to redistribute, *TI* Time interval

### Absence of cytoplasmic halo was associated with a decreased live birth rate after fresh cleaved embryo transfer

The cycle characteristics and pregnancy outcomes after the cleaved embryo transfers were compared between the halo-positive and halo-negative groups (Table 1). Although the female age was comparable between the groups (*P* = 0.0517), the male age was significantly higher in the halo-negative group than in the halo-positive group (*P* = 0.0001). The number of previous embryo transfer cycles, infertility cause, ovarian stimulation, serum hormone level, number of oocytes retrieved, oocyte diameter, and semen quality were comparable between the groups. The morphological grade of the cleaved embryos on day 2 was poorer in the halo-negative group than in the halo-positive group (Table 1). The clinical pregnancy and ongoing pregnancy outcomes in the halo-negative group were similar to those in the halo-positive group (*P* = 0.1087 and *P* = 0.0600, respectively). However, the live birth rate was significantly lower in the halo-negative group than in the halo-positive group (*P* = 0.0233). An increased miscarriage rate was observed in the halo-negative group. Multivariate logistic regression analysis also revealed a decrease in the live birth rate in the halo-negative group (odds ratio 0.252; 95% CI 0.037–0.880; *P* = 0.0282; Table S2 in Additional file 1).

### A prolonged cytoplasmic halo adversely impacted the live birth rate after fresh cleaved embryo transfer

The association between the duration of the cytoplasmic halo and pregnancy outcomes was also assessed in the halo-positive group. Both, univariate (odds ratio 0.879; 95% CI 0.825–0.934; *P* <  0.0001) and multivariate (aOR 0.879; 95% CI 0.817–0.946; *P* = 0.0004) logistic regression analysis showed that a prolonged cytoplasmic halo during fertilisation significantly decreased the live birth rate after fresh cleaved embryo transfer on day 2 (Table 2). To examine which event during the presence of a cytoplasmic halo was associated with the clinical outcome, the duration of the halo was divided into three phases: TI from tHa to tHc, TI from tHc to tHr, and TI from tHr to tHd. Although the TI from tHa to tHc did not correlate with the live birth rate, prolongation of the TI from tHc to tHr (aOR 0.893; 95% CI 0.825–0.966; *P* = 0.0044) and the TI from tHr to tHd (aOR 0.784; 95% CI 0.607–0.985; *P* = 0.0356) was significantly correlated with a decreased live birth rate (Table S3 in Additional file 1).

**Table 2:** Logistic regression analysis of TI from tHa to tHd and live birth rate after fresh cleaved embryo transfer

	Univariate logistic regression analysis				Multivariate logistic regression analysis			
	OR	95% CI	*P*-value	AUC	aOR	95% CI	*P*-value	AUC
Female age	0.832	0.796–0.868	< 0.0001	0.704	0.827	0.778–0.878	< 0.0001	0.732
Male age	0.930	0.901–0.959	< 0.0001	0.611	1.018	0.978–1.060	0.3681	
Ovarian stimulation				0.594				
Natural	Reference	–			Reference	–		
Clomiphene citrate	0.401	0.263–0.618	< 0.0001		0.732	0.455–1.177	0.1979	
Letrozole	1.250	0.657–2.360	0.4938		1.046	0.526–2.077	0.8987	
Insemination method				0.506				
cIVF	Reference	–			Reference	–		
ICSI	0.943	0.666–1.347	0.7472		0.927	0.629–1.363	0.6990	
Blastomere number	1.081	0.986–1.185	0.0983	0.535	1.100	0.997–1.214	0.0575	
Morphological grade				0.550				
1	Reference	–	–		Reference	–	–	
2	1.212	0.731–2.049	0.4587		1.509	0.867–2.625	0.1452	
3	0.986	0.618–1.614	0.9560		1.138	0.681–1.899	0.6214	
4	0.981	0.049–6.974	0.9869		0.754	0.103–5.734	0.1656	
TI from tHa to tHd	0.879	0.825–0.934	< 0.0001	0.593	0.879	0.817–0.946	0.0004	

*aOR* Adjusted odds ratio, *AUC* Area under the curve, *CI* Confidence interval, *cIVF* Conventional in vitro fertilisation, *ICSI* Intracytoplasmic sperm injection, *OR* Odds ratio, *tHa* Time of halo initiation, *tHd* Time when the halo phenomenon disappeared, *TI* Time interval

### Factors potentially affecting the presence and duration of the cytoplasmic halo

Correlations between the cytoplasmic halo and patient and embryonic characteristics were also sought to identify further factors possibly associated with the characteristics of the cytoplasmic halo (Tables 3 and 4). The duration of the cytoplasmic halo was not correlated with female age, male age, hormone level, number of oocytes retrieved, or semen quality (Table 3), but was significantly associated with oocyte diameter, especially the TI from tHc to tHr and the TI from tHr to tHd (*P* = 0.0006 and *P* = 0.0009, respectively). The presence and duration of a cytoplasmic halo were not affected by the ovarian stimulation method used and the insemination method used (Table 4). However, more detailed analysis showed that the TI from tHa to tHc for ICSI-derived fertilised oocytes was shorter than that for conventional IVF-derived fertilised oocytes (*P* = 0.0122). However, the TI from tHc to tHr was longer in the ICSI group than in the conventional IVF group (*P* = 0.0121). The initial position of the male pronucleus was not correlated with the presence of a cytoplasmic halo, but was significantly associated with the duration of this cytoplasmic halo event; the TI from tHa to tHc was lengthened and the TI from tHc to tHr was shortened when the male pronucleus appeared in the cortical region. We also analysed the associations of the duration of the cytoplasmic halo with the positions of the male pronucleus and the halo. When the cytoplasmic halo appeared on the side opposite to that of the male pronucleus, the TI from tHc to tHr was significantly shortened (*P* = 0.0004); in contrast, the position of the female pronucleus in relation to the cytoplasmic halo was not associated with the duration of the halo. Multivariate analysis of the TI from tHc to tHr was performed to adjust for potential statistically confounding biases (Table S4 in Additional file 1). Multiple linear regression analysis revealed that the TI from tHc to tHr was significantly affected by the oocyte diameter (*P* = 0.0007), position of the male pronucleus (*P* = 0.0002), and position of male PN appearance over cytoplasmic halo appearance (*P* = 0.0280).

**Table 3:** Spearman correlation of duration of halo with serum hormone level, oocyte diameter, and semen quality

	TI from tHa to tHd	TI from tHa to tHc	TI from tHc to tHr	TI from tHr to tHd
Female age	0.0049 (*P* = 0.8832)	0.0350 (*P* = 0.2904)	−0.0312 (*P* = 0.3454)	0.0182 (*P* = 0.5823)
Male age	− 0.0557 (*P* = 0.0925)	−0.0382 (*P* = 0.2478)	− 0.0441 (*P* = 0.1829)	0.0354 (*P* = 0.2844)
Oestradiol^a^	0.0212 (*P* = 0.5339)	0.0231 (*P* = 0.4947)	0.0106 (*P* = 0.7555)	−0.0365 (*P* = 0.2827)
Progesterone^a^	0.0177 (*P* = 0.6028)	0.0020 (*P* = 0.9526)	0.0420 (*P* = 0.2170)	−0.0133 (*P* = 0.6968)
Number of oocytes retrieved	0.0298 (*P* = 0.3804)	0.0364 (*P* = 0.2850)	0.0194 (*P* = 0.5682)	0.0206 (*P* = 0.5450)
Oocyte diameter	−0.1888 (*P* < 0.0001)	0.0054 (*P* = 0.8734)	−0.1158 (*P* = 0.0006)	−0.1130 (*P* = 0.0009)
Total sperm number	0.0109 (*P* = 0.7505)	0.0141 (*P* = 0.6797)	0.0131 (*P* = 0.7013)	−0.0140 (*P* = 0.6822)
Motile sperm number	0.0019 (*P* = 0.9548)	0.0148 (*P* = 0.6651)	0.0012 (*P* = 0.9711)	−0.0191 (*P* = 0.5780)
Motile sperm rate	0.0036 (*P* = 0.9174)	0.0275 (*P* = 0.4222)	−0.0153 (*P* = 0.6555)	−0.0260 (*P* = 0.4486)
Abnormal sperm number	0.0072 (*P* = 0.8340)	0.0157 (*P* = 0.6465)	0.0106 (*P* = 0.7564)	−0.0152(*P* = 0.6573)
Abnormal sperm rate	− 0.0292 (*P* = 0.3937)	−0.0411 (*P* = 0.2194)	0.0378 (*P* = 0.2692)	−0.0011 (*P* = 0.9745)

^a^Serum hormone levels on day of trigger

*tHa* Time of halo initiation, *tHc* Time when centripetal movement ended, *tHd* Time when the halo phenomenon disappeared, *tHr* Time when the granules started to redistribute, *TI* Time interval

**Table 4:** Association of the cytoplasmic halo with type of ovarian stimulation

	Embryos with cytoplasmic halo, n (%)	TI from tHa to tHd	TI from tHa to tHc	TI from tHc to tHr	TI from tHr to tHd
**Ovarian stimulation**					
Natural	685 (95.9)	14.1 ± 0.2	5.4 ± 0.2	6.9 ± 0.3	1.7 ± 0.1
Clomiphene citrate	61 (95.3)	14.2 ± 0.1	5.7 ± 0.1	6.8 ± 0.1	1.8 ± 0.0
Letrozole	120 (96.8)	13.4 ± 0.4	5.8 ± 0.3	6.0 ± 0.4	1.6 ± 0.1
*p*-value	0.8694	0.0806	0.3142	0.0739	0.3542
**Insemination method**					
cIVF	266 (95.3)	14.1 ± 0.2	5.9 ± 0.1	6.4 ± 0.2	1.8 ± 0.1
ICSI	600 (96.3)	14.2 ± 0.1	5.5 ± 0.1	6.9 ± 0.1	1.7 ± 0.0
*p*-value	0.4926	0.6634	0.0122	0.0121	0.5077
**Initial position of the male PN**					
Central	215 (95.6)	14.1 ± 0.2	5.1 ± 0.1	7.6 ± 0.2	1.7 ± 0.1
Intermediate	265 (95.7)	14.1 ± 0.2	5.6 ± 0.1	6.7 ± 0.2	1.8 ± 0.1
Cortical	386 (96.5)	14.0 ± 0.1	6.0 ± 0.1	6.3 ± 0.1	1.7 ± 0.0
*p*-value	0.7959	0.4239	< 0.0001	< 0.0001	0.2038
**Male PN appearance over halo appearance**					
PN on same side (*n* = 165)	N.A.	14.8 ± 0.1	5.6 ± 0.2	7.4 ± 0.2	1.7 ± 0.1
PN on opposite side (*n* = 701)	N.A.	14.0 ± 0.2	5.7 ± 0.2	6.6 ± 0.1	1.7 ± 0.0
*p*-value	–	0.0011	0.7225	0.0004	0.9623
**Female PN appearance over halo appearance**					
Same side (*n* = 401)	N.A.	14.0 ± 0.1	5.6 ± 0.1	6.7 ± 0.1	1.7 ± 0.0
Opposite side (*n* = 465)	N.A.	14.3 ± 0.1	5.7 ± 0.2	6.8 ± 0.1	1.8 ± 0.0
*p*-value	–	0.0948	0.3170	0.5903	0.2471

The data are shown as the mean and standard error of mean unless otherwise indicated

*tHa* Time of halo initiation, *tHc* Time when centripetal movement ended, *tHd* Time when the halo phenomenon disappeared, *tHr* Time when the granules started to redistribute, *TI* Time interval, *cIVF* Conventional in vitro fertilisation, *ICSI* Intracytoplasmic sperm injection, *PN* Pronucleus, *N.A* Not applicable

## Discussion

Although several studies have demonstrated that nuclear events during the fertilisation process can predict clinical outcomes [23–26], the significance of cytoplasmic events during fertilisation has received little attention in clinical embryology. In this study, we have demonstrated that the presence and duration of the cytoplasmic halo predicts the likelihood of a live birth after fresh cleaved embryo transfer on day 2. Furthermore, we found that the characteristics of the cytoplasmic halo during fertilisation are affected by male age, oocyte diameter, and the position of the male pronucleus.

In a previous study, we demonstrated that the rate of development to the blastocyst stage and the ongoing pregnancy outcome after frozen blastocyst transfer could be predicted by assessing the morphokinetics of the cytoplasmic halo at the one-cell stage using time-lapse technology [14]. Therefore, we hypothesised that the cytoplasmic halo may be associated with the pregnancy outcome after fresh cleaved embryo transfer. The present study found that absence of a cytoplasmic halo adversely impacted the live birth outcome after embryo transfer on day 2. Furthermore, a prolonged cytoplasmic halo was correlated with a decrease in the likelihood of a live birth outcome. In particular, a longer TI from tHc to tHr, longer time taken for central repositioning of cytoplasmic granules, and a longer TI from tHr to tHd (i.e., time required for redistribution of cytoplasmic granules) resulted in a lower live birth rate. These findings support our hypothesis that the cytoplasmic halo can be used to predict the chances of a live birth after fresh cleaved embryo transfer. However, the biological significance of absence of a halo and prolonged movement of cytoplasmic granules is unclear. Therefore, the reason why the presence and duration of a cytoplasmic halo is predictive of the pregnancy outcome is still unexplained.

To further clarify the cytoplasmic halo during fertilisation, we examined the association between the characteristics of the halo and cycle/patient characteristics. Previous reports have identified some morphokinetic parameters that could be affected by the protocols used for ovarian stimulation [27, 28]. Although three types of minimal ovarian stimulation (natural, CC, and letrozole cycles) were included in this cohort, there is no evidence that minimal ovarian stimulation affects the presence or duration of the cytoplasmic halo. Furthermore, the characteristics of the cytoplasmic halo were not altered by serum oestradiol/progesterone levels on the day of the trigger or the number of oocytes retrieved. However, it is still unknown whether controlled ovarian stimulation using exogenous gonadotropins affects the characteristics of the cytoplasmic halo; therefore, further studies are needed to examine the effects of exogenous gonadotropins on the halo.

In this study, oocyte diameter was significantly associated with the duration of the cytoplasmic halo. Previous studies have found that oocyte diameter depended on the degree of growth and maturity and correlated with the morphological grade of human blastocysts, in that very small oocytes resulted in decreased good-quality blastulation rates [29, 30]. The halo is considered a marker of microtubule-organised translocation of cytoplasmic components to the centre of the oocyte [31]. Therefore, in small oocytes, the organisation of the cytoskeleton during growth and maturation of the cytoplasm is insufficient; this may lead to prolongation of the cytoplasmic halo. Furthermore, we found that male age was associated with the absence of a cytoplasmic halo, and that the position of the male pronucleus significantly affected the duration of the halo. Although univariate analysis demonstrated a significant correlation between the insemination method used and duration of the halo, this correlation was not found in multivariate analysis, suggesting that the position of the male pronucleus had more influence than the insemination method in this regard. When the male pronucleus appeared in the cortical region of the oocyte, the TI from tHa to tHc was lengthened and the TI from tHc to tHr was shortened. This finding indicates that the cytoplasmic halo is involved in movement of the pronucleus towards the centre of the oocyte. Moreover, the duration of the cytoplasmic halo, especially the TI from tHc to tHr, was significantly affected by the initial positions of the male pronucleus and halo. Other researchers have reported observing a cytoplasmic wave, that appears as a well-coordinated radial wave originating from the initial position of the male pronucleus and extends towards the periphery of the oocyte [13]; they suggested that this cytoplasmic wave is a morphokinetic manifestation of formation of the sperm aster by microtubules. Although we could not obtain accurate data for all fertilised oocytes because of technical difficulties, we observed one very consistent example of this manifestation, namely, the cytoplasmic wave appeared at the initial position of the male pronucleus and extended towards the opposite side of the ooplasm, as previously reported [13]; in addition, the cytoplasmic halo was initiated on the side opposite to the initial position of the cytoplasmic wave and appearance of the male pronucleus (Video S1 in Additional file 2). However, a different phenomenon was seen in some fertilised oocytes, whereby the cytoplasmic halo appeared on the same side as the initial position of the cytoplasmic wave (Video S2 in Additional file 3). In this case, the cytoplasmic wave tended to be partially extended in the ooplasm; this may have led to prolongation of the cytoplasmic halo. Therefore, the cytoplasmic wave and halo are likely to be an interlocked phenomenon, that promotes movement of the pronuclei and cytoplasmic organelles to complete the fertilisation events and the first cytokinesis.

This study has several limitations. First, the data were obtained from a single-centre cohort of patients; multicentre studies are now required to determine whether our findings can be generalised to other clinics with different protocols and/or patient demographics. Second, only natural or minimal stimulation cycles were used in this study. Therefore, our results need to be confirmed in a cohort with controlled ovarian hyperstimulation. Furthermore, in the present study, only 36 women were observed after the transfer of halo-negative embryos; this extremely low number may have influenced the results of the statistical analysis. Observational and molecular studies that examine the correlation between the cytoplasmic wave (the sperm aster) and the cytoplasmic halo are also required in the future to determine the exact biological significance of the cytoplasmic halo.

## Conclusions

Absence and prolongation of the cytoplasmic halo during fertilisation were negatively correlated with the live birth rate after fresh cleaved embryo transfer on day 2. The characteristics of the cytoplasmic halo were strongly associated with oocyte diameter and paternal factors, including male age and the initial position of the male pronucleus. These findings indicate that the morphokinetic parameters of the cytoplasmic halo during fertilisation can be used to refine the selection of more competent embryos for transfer at the cleavage stage.

## Supplementary Information

**Additional file 1: Table S1.** Characteristics of embryos. **Table S2.** Logistic regression analysis of presence of cytoplasmic halo and live births after fresh-cleavage embryo transfers. **Table S3.** Correlation of different time intervals with live births after fresh cleaved embryo transfers. **Table S4.** Multiple linear regression coefficients for time interval from tHc to tHr during fertilisation.

**Additional file 2: Video S1.** Typical morphogenetics of the cytoplasmic wave, appearance of the pronucleus, and appearance of the cytoplasmic halo.

**Additional file 3: Video 2.** Atypical morphogenetics of the cytoplasmic wave, appearance of the pronucleus, and appearance of the cytoplasmic halo.

## Data Availability

The primary data for this study is available from the authors on direct request.

## Abbreviations

CC: Clomiphene citrate
HTF: Human tubal fluid
ICSI: Intracytoplasmic sperm injection
IVF: In vitro fertilisation
TI: Time interval
