# Supplementary material for: Characteristics of the cytoplasmic halo during fertilisation correlate with the live birth rate after fresh cleaved embryo transfer on day 2 in minimal ovarian stimulation cycles: a retrospective observational study
Source: Reprod Biol Endocrinol. 2021 Nov 26;19:172. doi: 10.1186/s12958-021-00859-1 (PMC8620661; doi:10.1186/s12958-021-00859-1)
Supplement: Supplementary file 1 — Additional file 1: Table S1. Characteristics of embryos. Table S2. Logistic regression analysis of presence of cytoplasmic halo and live births after fresh-cleavage embryo transfers. Table S3. Correlation of different time intervals with live births after fresh cleaved embryo transfers. Table S4. Multiple linear regression coefficients for time interval from tHc to tHr during fertilisation. [file 12958_2021_859_MOESM1_ESM.docx]

**ADDITIONAL FILE 1**

**Table S1. Characteristics of embryos**

| Cleaved embryos analysed, n | 902 |
| --- | --- |
| Insemination method |  |
| Conventional in vitro fertilisation, n (%) | 279 (30.9) |
| Intracytoplasmic sperm injection, n (%) | 623 (69.1) |
| Cytoplasmic halo-positive embryos, n (%) | 866 (96.0) |
| Cytoplasmic halo-negative embryos, n (%) | 36 (4.0) |
| TI from tHa to tHc (h) | 5.66 ± 0.07 |
| TI from tHc to tHr (h) | 6.74 ± 0.09 |
| TI from tHr to tHd (h) | 1.73 ± 0.03 |
| TI from tHa to tHd (h) | 14.13 ± 0.09 |

The data are shown as the mean and standard error of mean unless otherwise indicated.

**Table S2. Logistic regression analysis of presence of cytoplasmic halo and live births after fresh-cleavage embryo transfers**

|  | Univariate analysis | | | |  | Multivariate analysis | | | |
| --- | --- | --- | --- | --- | --- | --- | --- | --- | --- |
|  | Odds ratio | 95% CI | *P*-value | AUC |  | aOR | 95% CI | *P*-value | AUC |
| Female age | 0.832 | 0.796–0.868 | < 0.0001 | 0.704 |  | 0.823 | 0.775–0.872 | < 0.0001 | 0.720 |
| Male age | 0.932 | 0.904–0.960 | < 0.0001 | 0.611 |  | 1.028 | 0.989–1.069 | 0.1624 |  |
| Ovarian stimulation |  |  |  | 0.591 |  |  |  |  |  |
| Natural | Reference | – |  |  |  | Reference | – |  |  |
| Clomiphene citrate | 0.409 | 0.269–0.627 | < 0.0001 |  |  | 0.743 | 0.465–1.189 | 0.2154 |  |
| Letrozole | 1.215 | 0.643–2.274 | 0.5457 |  |  | 1.137 | 0.583–2.217 | 0.7065 |  |
| Insemination method |  |  |  | 0.506 |  |  |  |  |  |
| cIVF | Reference | – |  |  |  | Reference | – |  |  |
| ICSI | 0.945 | 0.670–1.344 | 0.7515 |  |  | 0.930 | 0.637–1.358 | 0.7085 |  |
| Blastomere number | 1.080 | 0.985–1.185 | 0.0898 | 0.536 |  | 1.100 | 0.997–1.214 | 0.0575 |  |
| Morphological grade |  |  |  | 0.550 |  |  |  |  |  |
| Grade 1 | Reference | – | – |  |  | Reference | – | – |  |
| Grade 2 | 1.202 | 0.727–2.027 | 0.4582 |  |  | 1.456 | 0.473–1.252 | 0.2919 |  |
| Grade 3 | 0.996 | 0.626–1.625 | 0.9857 |  |  | 1.082 | 0.652–1.793 | 0.7595 |  |
| Grade 4 | 0.679 | 0.035–4.208 | 0.7140 |  |  | 0.743 | 0.143–9.737 | 0.4748 |  |
| Cytoplasmic halo |  |  |  | 0.523 |  |  |  |  |  |
| Positive | Reference | – | – |  |  | Reference | – | – |  |
| Negative | 0.220 | 0.035–0.730 | 0.0096 | 0.518 |  | 0.252 | 0.037–0.880 | 0.0282 |  |

aOR, adjusted odds ratio; AUC, area under the curve; CI, confidence interval; cIVF, conventional in vitro fertilization; ICSI, intracytoplasmic sperm injection

**Table S3. Correlation of different time intervals with live births after fresh cleaved embryo transfers**

|  | aOR | 95% CI | *P*-value | AUC |
| --- | --- | --- | --- | --- |
| TI from tHa to tHc | 0.938 | 0.857–1.024 | 0.1573 | 0.721 |
| TI from tHc to tHr | 0.893 | 0.825–0.966 | 0.0044 | 0.733 |
| TI from tHr to tHd | 0.784 | 0.607–0.985 | 0.0356 | 0.720 |

Confounders: female age, male age, ovarian stimulation, insemination method used, number of blastomeres, and morphological grade. aOR, adjusted odds ratio; AUC, area under the curve; CI, confidence interval; tHa, time of halo initiation; tHc, time when centripetal movement ended; tHd, time when the halo phenomenon disappeared; tHr, time when cytoplasmic granules started to redistribute; TI, time interval

**Table S4.** **Multiple linear regression coefficients for time interval from tHc to tHr during fertilisation**

|  | Regression coefficient | 95% CI | | Standard error | *t*-value | *P*-value |
| --- | --- | --- | --- | --- | --- | --- |
|  |  | Lower | Upper |  |  |  |
| Male age | -0.012 | -0.044 | 0.020 | 0.016 | -0.73 | 0.4676 |
| Insemination method cIVF | Reference | − | − | − | − | − |
| ICSI | 0.123 | -0.084 | 0.106 | 0.105 | 1.17 | 0.2430 |
| Oocyte diameter | -0.078 | -0.123 | -0.033 | 0.023 | -3.40 | 0.0007 |
| Position of male pronucleus on appearance |  |  |  |  |  |  |
| Central | Reference | − | − | − | − | − |
| Intermediate | -0.155 | -0.425 | 0.114 | 0.137 | -1.13 | 0.2581 |
| Cortical | -0.502 | -0.762 | -0.241 | 0.132 | -3.78 | 0.0002 |
| Position of male pronucleus in relation to cytoplasmic halo on appearance |  |  |  |  |  |  |
| Same side | Reference | − | − | − | − | − |
| Opposite side | -0.269 | -0.509 | -0.029 | 0.122 | -2.20 | 0.0280 |

CI, confidence interval; cIVF, conventional in vitro fertilisation; tHc, time when centripetal movement ended; tHr, time when cytoplasmic granules started to redistribute; ICSI, intracytoplasmic sperm injection
